# Investigating the Potential Plasticizing Effect of Di-Carboxylic Acids for the Manufacturing of Solid Oral Forms with Copovidone and Ibuprofen by Selective Laser Sintering

**DOI:** 10.3390/polym13193282

**Published:** 2021-09-26

**Authors:** Yanis Abdelhamid Gueche, Noelia M. Sanchez-Ballester, Bernard Bataille, Adrien Aubert, Jean-Christophe Rossi, Ian Soulairol

**Affiliations:** 1 ICGM, University Montpellier, CNRS, ENSCM, 34000 Montpellier, France; yanis-abdelhamid.gueche@etu.umontpellier.fr (Y.A.G.); noelia.sanchez-ballester@umontpellier.fr (N.M.S.-B.); bernard.bataille@umontpellier.fr (B.B.); adrien.aubert@umontpellier.fr (A.A.); 2 Department of Pharmacy, Nîmes University Hospital, 30900 Nimes, France; 3 IBMM, University Montpellier, CNRS, ENSCM, 34000 Montpellier, France; jean-christophe.rossi@umontpellier.fr

**Keywords:** selective laser sintering, di-carboxylic acids, plasticizers, solid oral forms, printability, heating temperature

## Abstract

In selective laser sintering (SLS), the heating temperature is a critical parameter for printability but can also be deleterious for the stability of active ingredients. This work aims to explore the plasticizing effect of di-carboxylic acids on reducing the optimal heating temperature (OHT) of polymer powder during SLS. First, mixtures of copovidone and di-carboxylic acids (succinic, fumaric, maleic, malic and tartaric acids) as well as formulations with two forms of ibuprofen (acid and sodium salt) were prepared to sinter solid oral forms (SOFs), and their respective OHT was determined. Plasticization was further studied by differential scanning calorimetry (DSC) and Fourier-transform infrared spectroscopy (FTIR). Following this, the printed SOFs were characterized (solid state, weight, hardness, disintegration time, drug content and release). It was found that all acids (except tartaric acid) reduced the OHT, with succinic acid being the most efficient. In the case of ibuprofen, only the acid form demonstrated a plasticizing effect. DSC and FTIR corroborated these observations showing a decrease in the glass transition temperature and the presence of interactions, respectively. Furthermore, the properties of the sintered SOFs were not affected by plasticization and the API was not degraded in all formulations. In conclusion, this study is a proof-of-concept that processability in SLS can improve with the use of di-carboxylic acids.

## 1. Introduction

In the era of patient-centred medicine, the medical field is increasingly gaining interest in 3D printing [1,2]. This flexible technology enables manufacturing of complex structures that could match the anatomical and physiological needs of each patient [3]. For example, “Invisalign^®^” is a transparent 3D-printed orthodontic device that tailors the malocclusions of patients [4]. Recently, this innovative technology has expanded to the production of solid oral forms (SOFs) and the first Food and Drug Administration (FDA)-approved 3D printed pill, “Spritam^®^”, was commercialized in 2015 [5]. Among the different 3D printing techniques, selective laser sintering (SLS) shows great potential to produce personalized pharmaceutical oral forms [6,7], especially porous forms such as orally disintegrating printlets (ODPs) [8,9]. 

SLS is a 3D printing process based on the consolidation of powder particles by selectively scanning them with a laser, such as a CO_2_ laser [6]. While studies have already demonstrated the benefits of SLS to tune drug release by varying the structure of the printlets [10,11,12], there is still a lack of insight into the printability of pharmaceutical materials. Thus, before exploring the endless possibilities of pharmaceutical applications with SLS, it seems necessary to master the 3D printing process. Now, efforts are being performed by pharmaceutical engineering and formulation specialists to understand the relationship between the feedstock material and the process parameters [13,14]. In SLS, prior to sintering, the powder must be heated, and this heating temperature depends on the thermal properties of the polymer (glass transition temperature and melting temperature for amorphous and crystalline polymers, respectively) [15]. Optimal heating avoids phenomena such as curling of the sintered layers due to a high thermal gradient between the high laser energy and the temperature of the powder bed [16]. Furthermore, when the powder contains more than one component, the heating temperature that ensures optimal printing can change, especially if the new component modifies the thermal properties of the mixture. For instance, it has been demonstrated that paracetamol can reduce the heating temperature of the sintering process due to its plasticizing effect [17]. Similar observations were made in hot-melt extrusion (HME) [18] and fused deposition modelling (FDM) [19], where the introduction of plasticizers or even active pharmaceutical ingredients (APIs) decreased the extrusion and printing temperatures by reducing the melt viscosity of the formulation.

In SLS, most of the energy required to consolidate the powder is provided by the heating step, and then the laser finalizes the particle coalescence to produce the printlet [20]. Plasticizers could be beneficial in lowering the heating temperature, which would protect the API from degradation. Besides thermal degradation, studies have demonstrated that recycling unsintered powder that has undergone multiple heating cycles can modify the physicochemical properties of the material, such as particle size and molecular weight [21]. Therefore, plasticizers could be used as stabilizers to conserve the initial properties of the powder by decreasing the processing temperatures. 

Plasticizers are widely used in industrial manufacturing of plastics to improve the mechanical performances of the materials [22]. They are small molecules that increase the mobility of the polymer chains they are mixed with, usually by forming hydrogen bonds with the functional groups of the polymer [23]. While phthalates are one of the most frequently consumed plasticizers for the manufacturing of flexible plastics [24], they have recently been the subject of increasing concern because of their ubiquity in the environment [25] and their endocrine disrupting effects [26]. For these reasons, synthetic plasticizers are starting to be replaced by safer and more environmentally friendly plasticizers. Among these renewable and bio-sourced plasticizers are di-carboxylic acids such as succinic acid [27], fumaric acid [28] and tartaric acid [29]. Although these components are typically utilized as esters to plasticize thermoplastic polymers [30,31], they can also be used in their acid form [32]. 

There are several theories that explain the mechanisms behinds plasticization [33]. An example is the lubricity theory which holds that the plasticizer acts as a molecular lubricant under pressure, allowing the polymer chains to move freely [34]. In the gel theory [35], the plasticizer moves the attraction points (Van der Waals and hydrogen bonds) between the chains of the polymer which is considered as a gel, thereby increasing the mobility. The mechanistic theory is a modified version of the gel theory [36], stipulating that the plasticizer can move freely between the polymer chains by solvation / desolvation and are not fixed attraction points as suggested by the gel theory. On the other hand, the free volume theory considers that the plasticizer contributes to increase the free volume of the polymer and reduce the glass transition temperature (T_g_) [37], hence providing the chains more space to move. 

**Figure 1:**
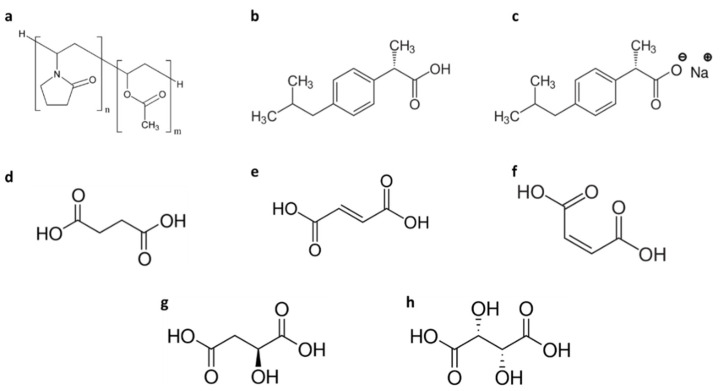
Structures of (**a**) copovidone KVA64, (**b**) ibuprofen acid, (**c**) ibuprofen sodium salt, (**d**) succinic acid, (**e**) fumaric acid, (**f**) maleic acid, (**g**) L-malic acid, (**h**) L-tartaric acid.

Although the plasticizing effect is well-known, the use of pharmaceutical excipients such as di-carboxylic acids to reduce the sintering temperature of the SLS process has not yet been explored. Thus, this work aims to investigate, as a proof of concept, the potential plasticizing effect of five structurally different di-carboxylic acids (Figure 1) by formulating them with copovidone (Kollidon VA64), one of the most investigated thermoplastic polymers in SLS for the manufacturing of solid oral forms (SOFs) [8,9,11]. As this copolymer of polyvinyl pyrrolidone and vinyl acetate only contains H-bond acceptors [38], a possible plasticizing effect can be envisaged with H-bond donors such as di-carboxylic acids. In addition, the effect of API as a plasticizer was further investigated in the case of ibuprofen. This API was chosen because its acid form possesses a carboxylic acid group that is available to act as H-bond donor as previously reported [39]. It is also a good example of a thermolabile drug with low melting and degradation temperatures which can undergo degradation when submitted to thermal stress [40]. Lastly, differential scanning calorimetry (DSC) and Fourier-transform infrared spectroscopy (FTIR) were conducted to explore the mechanisms of plasticization and the degradation of the drug was assessed by ultra-high performance liquid chromatography (UHPLC). 

## 2. Materials and Methods

### 2.1. Materials

Kollidon^®^ VA64 (KVA64) was donated by BASF (Ludwigshafen, Germany). Ibuprofen acid (IbuAc) and ibuprofen sodium salt anhydrous (IbuNa) were provided by Fagron (Rotterdam, Netherlands) and Sigma-Aldrich (Saint-Louis, MO, USA), respectively. Succinic acid (SA) (anhydrous, free-flowing, Redi-Dri™, ACS reagent ≥ 99.0%), fumaric acid (FA) (≥99.0% (T)), maleic acid (MA) (ReagentPlus^®^ ≥ 99% (HPLC)), L-(-)-malic acid (MLA) (≥95% (titration)) and L-(+)-tartaric acid (TA) (ACS reagent ≥ 99.5%) were purchased from Sigma-Aldrich (Saint-Louis, MO, USA). Figure 1 illustrates the chemical structure of the different components used in the printing process. 

Hydrochloric acid (37%) for the preparation of dissolution medium was purchased from Carlo Erba Reagents (Milano, Italy). Formic acid (reagent grade, ≥95%) and acetonitrile (HPLC gradient grade, ≥99%) were obtained from Sigma-Aldrich (Saint-Louis, MO, USA). 

### 2.2. Preparation of Mixtures

Binary mixtures of KVA64/di-carboxylic acid and KVA64/drug as well ternary mixtures of copovidone/succinic acid/IbuNa were prepared. Table 1 summarizes the composition of the different mixtures. Prior to mixing, due to their large particle size, the di-carboxylic acids were ground with a pestle and mortar, then all components were sieved through a 250 µm sieve. The mixing was carried out on a 3D shaker mixer Turbula® T2F (WAB, Muttenz, Swizterland) at a speed of 49 rpm for 10 min.

### 2.3. Printing of Solid Oral Forms

First, OnShape® (Onshape, Boston, MA, USA), an online computer-aided design (CAD) software and Slic3r^®^ 1.2.9, an open-source software, were used for the design and slicing of the cylindrical SOF (10 mm diameter × 3 mm height), respectively. Then, 300 g of each powder was loaded into the Sharebot^®^ SnowWhite 3D SLS printer (Sharebot, Nibionno, Italy) and thirty-six SOFs were launched for batch printing. Before printing, the powder was heated during 30 min and the temperature mode was set to *powder temperature*. While the laser power, scan speed and layer thickness were set constant (25%, 35,000 pps and 100 µm, respectively), the powder temperature was modified as this is a key parameter for this study. It was first set to a low value empirically based on the glass transition temperature (T_g_) of the powder mixture and then incremented by 5 °C until the optimal heating temperature was attained (Table 2). The optimal heating temperature (OHT) corresponded to the minimum temperature at which all SOFs were completely printed without curling of the sintered layers in a reproducible manner (three times). 

After sintering with a CO_2_ laser (λ = 10.6 µm), SOFs were removed from the printing bed and brushed to remove their powder excess. 

### 2.4. Differential Scanning Calorimetry (DSC) 

DSC was used to determine the melting point (or glass transition temperature) of the individual components (copovidone, drugs and di-carboxylic acids) and the different physical mixtures prepared. Samples of 5–10 mg were placed in sealed aluminum pans and heated from 25 °C to 200 °C at 10 °C/min with a DSC 4000 (Perkin Elmer, Waltham, MA, USA). A heat-cool-heat cycle method was used to remove the thermal history of copovidone. Nitrogen was employed as the purge gas with a flow rate of 20 mL/min. Data collection and analysis were carried out with Pyris Manager software (Perkin Elmer, Waltham, MA, USA). The glass transition temperature measurements were realized in triplicate and the results were expressed as the mean value ± standard deviation.

### 2.5. Thermogravimetric Analysis (TGA)

TGA was used to characterize the degradation profile and determine the 2% degradation point (Td_2%_) of the different components (copovidone, drugs and di-carboxylic acids). The measurements were performed with a TGA Q50 (TA instruments, Waters Corporation, New Castle, DE, USA) from 30 °C to 700 °C at a heating rate of 15 °C/min under air flow. Platinum pans were used with an average sample weight of 10 mg. Data analysis was conducted using Universal Analysis 2000 (TA instruments, Waters Corporation, New Castle, DE, USA).

### 2.6. Fourier-Transform Infrared Spectroscopy (FTIR) 

Infrared spectrophotometer Vector 22 FTIR (Bruker, Billerica, MA, USA) was employed to investigate potential hydrogen bond interactions formation during SLS. The absorbance of individual components (copovidone, drugs and di-carboxylic acids) and sintered SOFs was recorded from 4000 to 400 cm^−1^ at room temperature and averaged over 32 scans at 2 cm^−1^ resolution. Disks of 100 mg were prepared by mixing and then compressing 10 mg of the sample (or 0.5 mg for the drugs and the di-carboxylic acids) with Q.S. (Quantum satis) of anhydrous potassium bromide (previously dried in the oven at 100 °C for 30 min). The FTIR spectrums were treated using OPUS 6.5 infrared software (Bruker, Billerica, MA, USA).

### 2.7. X-ray Powder Diffraction (XRPD)

The solid state of the materials used in this study and the sintered SOFs was characterized using a Bruker D8 Advance diffractometer (Bruker, Billerica, MA, USA) and monochromatic Cu Kα1 radiation (λα = 1.5406 Å, 40 kV and 40 mA). The angular range of data recorded was 2–70° 2Ɵ, with a stepwise size of 0.02° and a speed of 0.1 s counting time per step, using LINXEYE detector 1D.

### 2.8. Weight, Hardness and Disintegration Time of the Sintered SOFs

For each formulation, the weight and hardness of ten SOFs were determined using an Adventurer^®^ precision electronic balance (OHAUS, Parsippany, NJ, USA) and a Sotax Multitest 50FT (Sotax AG, Switzerland), respectively. 

Disintegration tests were performed on a Sotax DT50 disintegration apparatus (Sotax AG, Switzerland) with distilled water (800 mL) at 37 °C following the European Pharmacopeia guidelines [41]. For each powder, six SOFs were tested simultaneously. The disintegration time was reached when no residue was present at the bottom of the test basket. The results were reported as a mean value ± standard deviation.

### 2.9. Drug Release of the Sintered SOFs

A dissolution test was carried out for SOFs containing IbuNa or IbuAc with a Pharma Test DT70 dissolution tester (Hainburg, Germany) using a paddle-type apparatus (European Pharmacopeia) [42]. For each formulation, three SOFs were individually placed in the dissolution vessels each containing 800 mL of 0.1 M HCl and stirred at 100 rpm and 37 ± 0.5 °C. Samples were automatically analyzed every 5 min using a continuous flow system connected to an 8 cell Specord 250 UV/Vis spectrophotometer (Analytik Jena, Germany) at a wavelength of 268 nm. The results were expressed as mean values ± standard deviation.

### 2.10. Drug Content of the Sintered SOFs

For each formulation, three SOFs were dissolved in 100 mL of a mixture (40%/60%) of solvent A (distilled water/formic acid (1%, *v/v*)) and solvent B (acetonitrile). Samples of the solutions were then diluted and the concentration of the drug was determined by ultra-high performance liquid chromatography (UHPLC) using a UHPLC-DAD system. This consisted of a Thermo Scientific™ Dionex™ UltiMate™ 3000 BioRS equipped with a WPS-3000TBRS autosampler and a TCC-3000RS column compartment set at 35 °C (Thermofisher Scientific, Waltham MA, USA). The system was operated using Chromeleon 7 software. An Accucore C18 column (2.6 µm, 100 mm × 2.1 mm) combined with a security guard ultra-cartridge (Phenomenex Inc., Torrance CA, USA) was used. An isocratic binary solvent system was utilized, consisting of solvent A and solvent B (40%A, 60%B). The flow rate of the mobile phase was 1.5 mL/minute, and the injection volume was 50 μL. Quantitative analysis of IbuAc and IbuNa in the SOFs was carried out using an external standard method. The calibration curve for each form of the drug was constructed using 5 different standard levels of the corresponding form in the concentration range 1–20 mg/L. The peak of ibuprofen was monitored at 258 nm. 

### 2.11. Statistical Analysis

The effect of formulation composition on the glass transition temperature, weight, hardness and disintegration time of SOFs was analyzed statistically. One-way analysis of variance (ANOVA) in conjunction with Tukey’s HSD (honestly significant differences) test were used to determine the statistical significance of the differences among the groups (*p* < 0.05).

## 3. Results and Discussion

For this study, different di-carboxylic acids were investigated as potential plasticizers. In order to assess how structural changes such as the presence of a double bond, orientation of the carboxylic acid groups in the molecule or the presence of hydroxyl groups can affect the plasticizing effect, succinic acid (butanedioic acid) was compared with fumaric ((2E)-but-2-enedioic), maleic ((2Z)-but-2-enedioic), malic (2-hydroxybutanedioic) and tartaric (2,3-dihydroxybutanedioic) acids. In addition, IbuAc and IbuNa were used to explore the potential effect on plasticization of switching an API from its acid form to its salt form. 

### 3.1. Thermal Analysis 

One of the most common ways to demonstrate a plasticizing effect is to analyse the samples by DSC and observe whether a decrease in the glass transition temperature (T_g_) of the polymer is detected [43]. In this study, T_g_ of the different mixtures were measured from the second heating scan after removal of their thermal history [17]. After the first heating scan, the components present in all formulations were dissolved in the polymeric matrix and amorphized, hence no endothermic melting peaks were observed. Therefore, DSC thermograms of the physical mixtures containing copovidone and drugs or acids revealed only the T_g_. Table 3 shows the glass transition temperatures of copovidone and the different prepared mixtures. 

Results in Table 3 show a decrease in the glass transition temperature of copovidone from 99.3 °C to 93.2 °C and 80.7 °C in the presence of IbuNa and IbuAc, respectively. The greater decrease in T_g_ observed with IbuAc may be due to the formation of hydrogen bonds between the copovidone and the carboxylic acid group of IbuAc which induces an increase in the mobility of the system [44]. Table 3 shows that adding the different di-carboxylic acids at rate of 5% to copovidone provokes a reduction in T_g_. On one hand, T_g_ decreased to 89.6 °C after TA was incorporated. On the other hand, mixtures with the other acids (SA, FA, MA and MLA) presented a lower and similar T_g_ (84.3–85.7 °C) with no statistically significant difference. In addition, as the proportion of succinic acid increased, the T_g_ reduced proportionally to 57.5 °C for 20% of SA. Furthermore, ternary mixtures containing copovidone, SA and IbuNa showed a lower T_g_ compared to their binary counterpart mixtures with the same percentage of SA, which could imply a synergistic effect of SA and IbuNa on plasticization. 

The advantage of using plasticizers is their ability to reduce the optimal heating temperatures (OHT). This effect can be evidenced by a solidification of the powder bed when processing the mixture at the same OHT for the polymer. This implies that the heating temperature is too high for the mixture as it exhibits a high flow and consolidates in a block before the laser even starts sintering. The heating should only approach the powder temperature to the point when the material starts to flow without inducing fluidization [45]. In contrast, when a powder containing a non-plasticizing component is processed at a temperature inferior to the OHT of the raw polymer, a curling typically occurs. 

Based on these observations, the OHT for the different powders were identified as the minimum heating temperature at which no curling or powder solidification were observed (Table 2). It can be pointed out that despite a decrease in the T_g_ when both IbuNa and TA were mixed with KVA64, the OHT of the mixture powders was not different from the OHT for pure KVA64. For the other acids (SA, FA, MA, MLA), as well as IbuAc, a decrease in OHT was observed (Table 2). While the addition of 5% of IbuAc favoured a decrease in OHT by 40 °C, a reduction of 15 °C was observed when 5% of SA was added. A decrease of only 5°C was observed after the addition of 5% of FA, MA or MLA. 

These differences demonstrate that di-carboxylic acids have a different effect on processability even though each of SA, FA, MA and MLA show a similar decrease in T_g_ during DSC analysis. Furthermore, as the percentage of SA increased, the OHT decreased further until it reached a minimum of 80 °C for 15% of SA (Table 2). Above this ratio, the OHT was not affected even though T_g_ continued to decrease at 20% of SA, demonstrating a saturation of plasticization at 15% of SA. This saturation effect has been previously reported with other plasticizers. For example, Aydin et al. demonstrated that increasing the mannitol ratio above 5% does not improve plasticization of starch films [46]. Moreover, IbuNa did not affect the processability when it was introduced in the mixtures of KVA64 and SA, as the OHT were the same for binary and ternary mixtures at an equivalent ratio of succinic acid (Table 2). Hence, no synergistic effect between IbuNa and succinic acid on processability was demonstrated.

**Figure 2:**
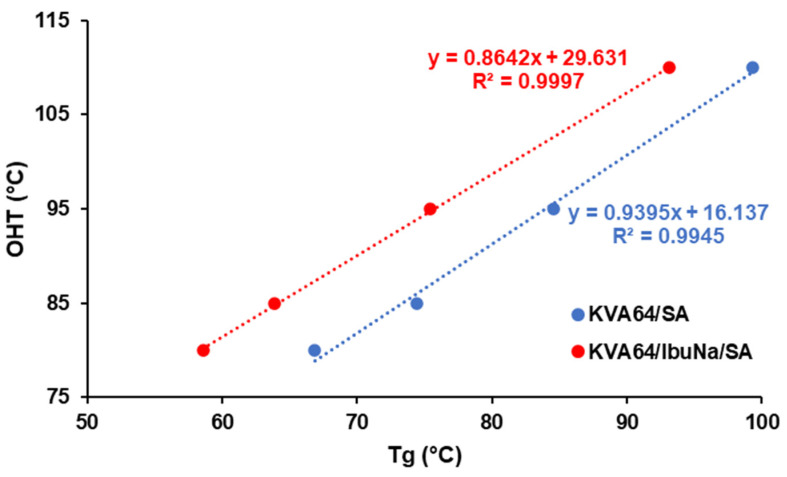
Relationship between glass transition temperatures and optimal heating temperatures at different ratios of succinic acid for mixtures of KVA64/SA and mixtures of KVA64/IbuNa/SA.

Among the different excipients used, SA demonstrated the highest plasticizing efficiency. Therefore, the linearity of the T_g_ and OHT of the binary and ternary mixtures at four different succinic acid ratios (0, 5, 10 and 15%) was evaluated in the same manner as in previous work with binary mixtures of KVA64 and paracetamol at different ratios [17]. Figure 2 confirms the existence of a linear relationship with high correlation coefficients (R^2^) 0.9945 and 0.9997 for both the binary and ternary mixtures, respectively. It is important to note that this linearity is not maintained above 15% of succinic acid. As the slope and the ordinate at the origin are not the same for both systems, it could be postulated that the linear regression equation relating T_g_ and OHT is only constant within a mixture (binary or ternary) of the same composition at different ratios of one component.

The method applied to measure the glass transition temperatures shows limits, as it requires, firstly, to be heated to 200 °C to remove the thermal history of KVA64, which exhibits an important endothermic peak at ~170–200 °C despite its amorphous state (Supplementary material: Figure S1). In contrast, the heating temperatures used during the SLS process did not exceed 110 °C (Table 2). Therefore, the plasticization that occurs during the first heating of DSC does not perfectly reproduce the interactions that take place during the SLS heating step. The higher temperatures used in DSC favour more contact between the small molecules and the polymer, and the generation of chemical interactions [47]. Hence, the measured glass transition temperatures may be overestimated. This could explain the reduction of T_g_ even with components that do not exhibit a plasticizing effect in SLS. However, these results are still of interest and serve as initial comparison of the effect of different plasticizers and ratios on the SLS printing temperature.

Table 3 shows that the melting of the different di-carboxylic acids and both forms of ibuprofen takes place at temperatures higher than the OHT of the respective mixtures. This confirms that powder consolidation during the heating step was not influenced by melting of the added excipients or drugs, but only dependant on the glass transition of the polymer as it accounts for most of the powder bed (75–95%). Furthermore, the solidification of an amorphous polymer depends on its glass transition temperature [15,16,48].

Temperatures for which 2% of the component was degraded were evaluated with TGA (Table 3). Copovidone degraded by 2% at 302 °C whereas Td_2%_ of the di-carboxylic acids were between 142 and 194 °C. Furthermore, IbuNa exhibited a higher thermal stability (Td_2%_ = 234 °C) compared to IbuAc (Td_2%_ = 137 °C). The Td_2%_ of the prepared mixtures (Table 3) were found to be inferior compared to pure copovidone due to the addition of more thermolabile drugs and di-carboxylic acids. The heating temperatures in SLS were largely below the degradation points of the different components, but the CO_2_ laser can also degrade them, particularly IbuAc. Thus, UHPLC assays were conducted to further investigate this. 

TGA analysis showed that the water content (WC%) of the different acids and IbuAc was inferior to 0.5%. Meanwhile, KVA64 and IbuNa showed high WC%: 2.35% and 14.66%, respectively. The anhydrous form of racemic (R,S)-(±)-ibuprofen sodium salt is highly unstable and converts rapidly to the dihydrate form when exposed to the humid environment [49], which explains the high water content corresponding to the water of crystallization. A dehydration endothermic peak was also observed in the DSC thermogram of IbuNa at 102.6 °C (Supplementary material: Figure S2). Despite the well-known plasticizing properties of water [50,51], it did not play a role in lowering the optimal heating temperature when it was bound to sodium salt ibuprofen. 

### 3.2. FTIR Analysis 

**Figure 3:**
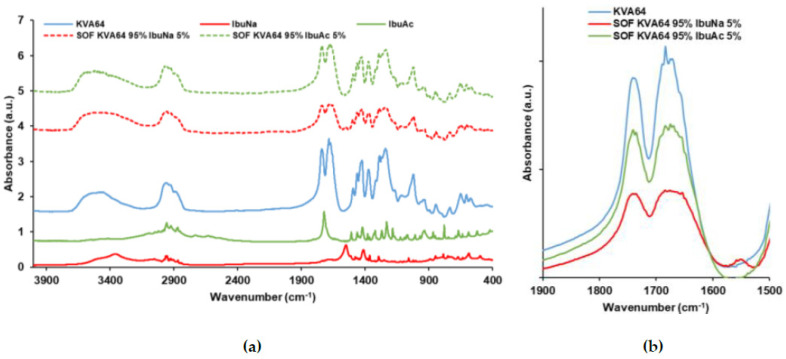
FTIR spectrums of KVA64, IbuNa, IbuAc and printed SOFs: (**a**) from 400 to 4000 cm^−1^, (**b**) from 1500 to 1900 cm^−1^.

FTIR was used to explore the potential formation of H-bonds between the different materials used in the formulations studied and to provide insight into the plasticization mechanisms that occur during the heating step in SLS. These bonds could form between the C=O groups of the copovidone and the OH groups of carboxylic acids, as previously demonstrated by Hurley et al. [52]. KVA64 has two strong main peaks at 1740 cm^−1^ (C=O stretch of the vinyl acetate) and 1683 cm^−1^ (C=O stretch of the tertiary amide) as shown in Figure 3a. The variation of these two peaks was studied as copovidone is the major component in the formulations used in this work. FTIR spectrum of APIs (Figure 3a) showed a peak at 1549 cm^−1^ for IbuNa due to the C=O stretch of the carboxylate group and a sharp peak at 1721 cm^−1^ for IbuAc corresponding to the C=O stretch of the carboxylic acid group. Both C=O peaks of KVA64 did not shift to lower or higher frequencies when the polymer was sintered with both drugs, but they broadened and decreased in intensity. The broadening was more pronounced with the tertiary amide C=O peak (1683 cm^−1^) and when IbuNa was introduced. This indicates the presence of interactions between the polymer and both drugs. 

**Figure 4:**
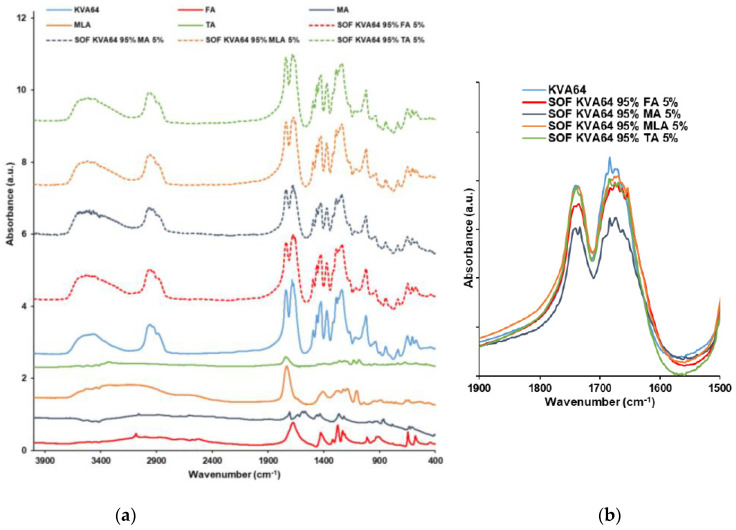
FTIR spectrums of KVA64, FA, MA, MLA, TA and printed SOFs: (**a**) from 400 to 4000 cm^−1^, (**b**) from 1500 to 1900 cm^−1^.

Fumaric acid and maleic acid were characterized by their C=O carboxylic stretch peaks at 1674 and 1706 cm^−1^, respectively (Figure 4a). Malic acid presents a strong peak at 1731 cm^−1^ due to its COOH group and a large band from 2700 to 3700 cm^−1^ due to its hydroxyl group (Figure 4a). Furthermore, tartaric acid shows a small C=O stretch peak at 1738 cm^−1^ and a large band from 2700 to 3700 cm^−1^ due to its two hydroxyl groups (Figure 4a). In the spectrums of SOFs, the peak of KVA64 at 1683 cm^−1^ broadened (Figure 4b), which could be due to the formation of H-bonds. These variations could also be explained by the overlapping of the C=O peaks of di-carboxylic acids with the characteristic peaks of KVA64. 

**Figure 5:**
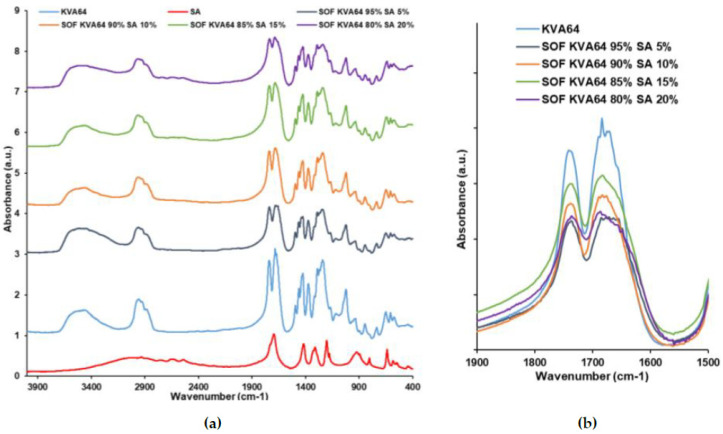
FTIR spectrums of KVA64, SA and printed SOFs: (**a**) from 400 to 4000 cm^−1^, (**b**) from 1500 to 1900 cm^−1^.

Succinic acid exhibited in FTIR a characteristic peak at 1681 cm^−1^ due to C=O stretch of its carboxylic group, which overlaps with the characteristic peaks of KVA64 (Figure 5a). FTIR spectra of SOFs prepared with different ratios of SA showed a broadening of the C=O amide peak of KVA64. This could be due to H-bond formation or overlapping of the bands of the two components. 

**Figure 6:**
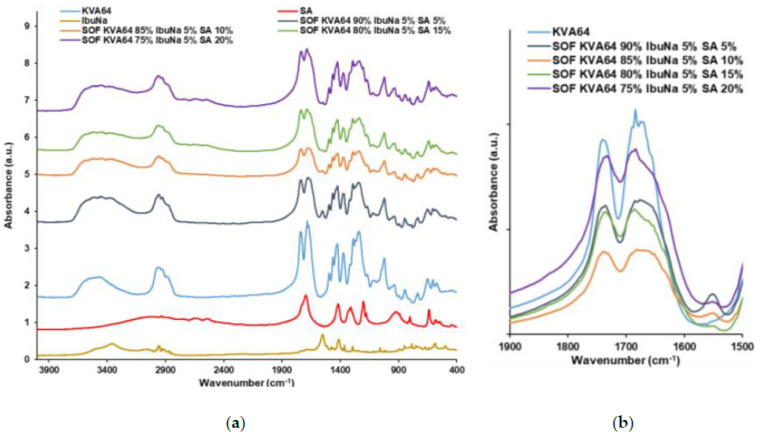
FTIR spectrums of KVA64, SA, IbuNa and printed SOFs: (**a**) from 400 to 4000 cm^−1^, (**b**) from 1500 to 1900 cm^−1^.

The FTIR spectra of the SOFs prepared with KVA64, IbuNa and SA also showed a broadening of the C=O amide peak of copovidone (Figure 6b). Notably, although the C=O stretch peak of IbuNa at 1549 cm^−1^ was found in all the SOFs, its intensity decreased with the proportion of succinic acid (Figure 6a), suggesting that a high content of SA promotes the amorphization of IbuNa.

Overall, all the SOFs prepared with mixtures of KVA64, di-carboxylic acids and/or drugs display interactions especially at the wavenumber 1683 cm^-1^, which corresponds to the C=O amide peak of copovidone. This group is more reactive and more susceptible to form H-bonds than the C=O vinyl acetate function, as previously reported by Yuan et al. [53]. Although no change in frequency has been observed, variations in the width and intensity could also be interpreted as the formation of intermolecular hydrogen bonds [46]. The decrease in the intensity and the width of the C=O amide peak may indicate that the interactions between the copovidone molecules are being replaced by interactions between the polymer and the other SOFs components. Nevertheless, the FTIR data did not allow differentiation between the distinct components in terms of plasticization. For instance, all of the di-carboxylic acids and drugs exhibited interactions, despite having a different effect on the optimal heating temperature. Moreover, as the proportion of succinic acid increased, there was no proportional broadening of the peak (Figure 5b and Figure 6b).

For the IbuNa, even with an absence of H-bond donors in its structure, interactions were found with the C=O group of KVA64. This may be due to the important moisture content present in the ibuprofen sodium salt that could participate in H-bond formation [54]. 

As noted by Matet et al. [55], FTIR is not a discriminatory technique and could not reveal differences between the different polymer/plasticizers mixtures. Moreover, the high hygroscopicity of copovidone [56] and the overlapping of the peaks are limits for the interpretation of FTIR results. H-bonds are temporary bonds that could evolve as function of the applied temperatures [57]. Hence, further work could involve conducting FTIR at the temperatures of the heating process [58] to mimic the interactions between the components under real sintering conditions. 

### 3.3. Solid State Analysis 

**Figure 7:**
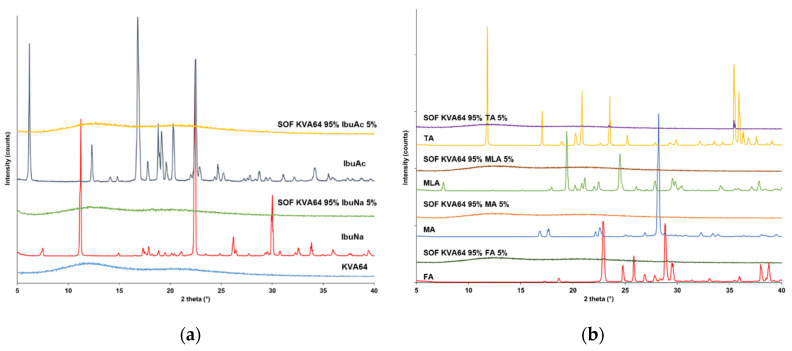

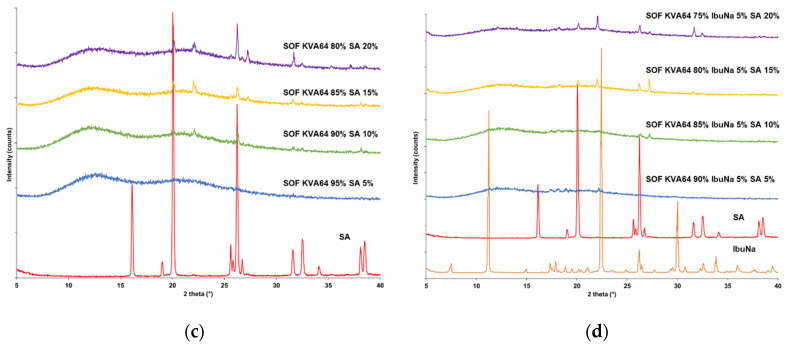
XRPD patterns: (**a**) KVA64, IbuNa, IbuAc and their sintered SOFs, (**b**) FA, MA, MLA, TA and their sintered SOFs, (**c**) SA and its sintered SOFs, (**d**) IbuNa, SA and their sintered SOFs.

XRPD analysis was carried out to study the solid state of the different components and the potential solid-state transitions due to the sintering process. All the used materials are crystalline except copovidone which is amorphous and did not expose any crystalline peaks on XRPD. X-ray diffractograms of drugs (Figure 7a) display the characteristic crystalline peaks of the racemic form for both ibuprofen acid and ibuprofen sodium salt dihydrate, in agreement with the literature data [49,59]. However, these crystalline peaks were absent in the diffractograms of the sintered SOFs (Figure 7a), indicating an amorphization of the drugs. Previous studies have already demonstrated that SLS could produce amorphous solid dispersions, due its two thermal steps (heating and laser scanning) [60,61]. 

Figure 7b exhibits the effect of sintering on the initially crystalline di-carboxylic acids. While MA and MLA underwent amorphization as demonstrated by the absence of peaks in the X-ray diffractograms of the SOFs prepared with these acids. For the case of SOFs prepared with FA and TA, some peaks were still distinguishable but with reduced intensity, suggesting a partial amorphization of the fumaric and tartaric acids. The differences observed in the rate of amorphization could be correlated with the melting points of the di-carboxylic acids. As FA and TA present the higher melting points, higher temperatures will be needed to dissolve completely in the polymeric matrix. 

X-ray diffractogram of the succinic acid (Figure 7c) shows characteristic peaks of the form ꞵ [62]. As previously seen for MA and MLA, no peaks were observed in the diffractogram of the SOF sintered with 5% of SA, implying an amorphization of the acid. However, the SOFs prepared with higher ratio of SA exhibited peaks at 20.1, 22.1, 26.2, 27.3, 31.6, 32.6° and their intensity increased proportionally with the percentage of succinic acid incorporated. It is interesting to note that two peaks (22.1 and 27.3°) did not appear in the diffractogram of pure SA. These two peaks are specific to the form α, which is favoured by high temperatures, as suggested by Yu et al. [62]. This implies that in addition to a partial amorphization of the succinic acid, sintering promoted the recrystallisation into the form α.

Regarding the SOFs prepared with the ternary mixture of copovidone, IbuNa and SA (Figure 7d), partial amorphization of the ꞵ-succinic acid and recrystallisation into the form α was also observed at the SA ratios of 10, 15 and 20%. As for the IbuNa, three small peaks characteristic of the API (17.5, 18.3, 19.0°) and two other peaks that were not in the diffractogram of the pure drug (22.3 and 22.6°) were present in the diffractogram of SOFs sintered with 5% of SA. This could be explained by the dehydration of the ibuprofen sodium salt dihydrate during the SLS process which could induce the rearrangement of the crystal structure as previously demonstrated by Censi et al. [49]. However, only the peak at 18.3° remained at high ratios of SA (15 and 20%). These results are in agreement with the FTIR observations, as the amorphization is stimulated at higher percentage of succinic acid. 

### 3.4. Characterisation of the Sintered SOFs 

The weight of the SOFs printed with the different powders varied between 129 and 170 mg (Table 4). The average weight of the SOFs decreased significantly with the introduction of IbuAc and increased significantly when IbuNa was incorporated. Among the different di-carboxylic acids, only SA and MA reduced the weight of SOFs significantly when they were incorporated. SOFs prepared with formulations of copovidone, SA and IbuNa exhibited a significant lower weight compared to the corresponding binary mixtures. However, increasing the succinic acid ratio did not show a significant influence on the weight variation. Table S1 (Supplementary material) shows that these variations in weight were not solely influenced by the powder compactness. This was confirmed by the observation of the SOF’s vertical sections by scanning electron microscopy (SEM) (Supplementary material: Figure S3). SOF printed with the mixture of KVA64 and the plasticizing SA showed a similar structure to the SOF produced with KVA64 and the non-plasticizing TA. Further work is ongoing to help understand better these observations.

Table 4 shows that all printed SOFs presented a low hardness (<40 N), which was expected because they were printed at a high scanning speed (35,000 pps) [8]. Forms printed only with KVA64 exhibited the higher hardness values (38.6 N). The mechanical properties of SOFs decreased significantly after the introduction of drugs, most importantly with IbuAc. As for the di-carboxylic acids, only SA, MA and MLA showed a significant negative effect on hardness. The hardness was not significantly affected by increasing the ratio of succinic acid, whether in the binary mixtures (KVA64/SA) or the ternary mixtures (KVA64/IbuNa/SA).

In contrast to other studies conducted on plasticization of polymers with di-carboxylic acids and their esters [31,32,63], this work does not show a clear link of plasticization to the reduction of the mechanical properties. For example, both succinic and maleic acid decreased the hardness by the same value with no significant difference despite having different plasticizing properties. Moreover, increasing the proportion of SA improved plasticization but did not deteriorate the hardness. Based on these observations, it can be concluded that the mechanical properties were mainly influenced by the proportion of copovidone in the powder mixtures. Indeed, a lower amount of Kollidon VA64 produces less sintered zones and more porosity [9,11], which could reduce the hardness of the SOFs. 

Table 4 shows that the longer disintegration time was observed for the SOFs sintered only with KVA64 (73 s). The introduction of other elements in the formulation reduced the disintegration time significantly below 60 s. The lowest values were observed for KVA64/IbuAc, KVA64/MA, binary KVA64/SA mixtures and ternary KVA64/IbuNa/SA mixtures, and no statistically significant difference was observed. The reduction in disintegration time could be correlated to the lower proportion of copovidone, which decreases the viscosity of the medium and slows down polymer erosion [17]. Overall, the SOFs disintegrated within the 3 min, which makes them suitable as orally disintegrating printlets according to the European Pharmacopeia [41]. 

**Figure 8:**
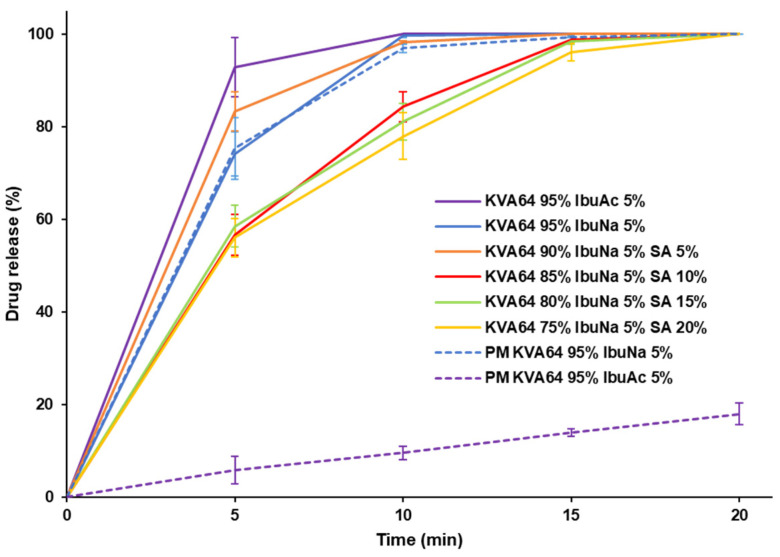
Dissolutions profiles of the different SOFs prepared with drugs and the physical mixtures of KVA64 95%/IbuNa 5% and KVA64 95%/IbuAc 5%.

Figure 8 shows the dissolution profiles of the different SOFs containing IbuNa and IbuAc. All formulations achieved more than 85% dissolution at 15 min, making them suitable for immediate release [64]. The formulation KVA64 95%/IbuNa 5% as well as KVA64 95%/IbuAc 5% exhibited 100% drug release at 10 min. The introduction of succinic acid at a high percentage (10, 15, 20%) in the formulation of KVA64 and IbuNa, slowed down drug release. This could be due to the acidification of the SOF’s microenvironment by the organic acid as previously mentioned by Sateesha et al. [65], which decreases the ionization of ibuprofen and hence its solubility. 

Figure 8 exhibits the differences in drug release between the physical mixtures and the prepared SOFs for both KVA64 95%/IbuNa 5% and KVA64 95%/IbuAc 5%. It can be noted that while amorphization induced by SLS did not influence the solubility of IbuNa, which is already high prior to sintering, the solubility of IbuAc significantly improved. At 20 min, full dissolution of the SOF was achieved whereas the physical mixture of IbuAc/KVA64 dissolved only up to 18%. This confirms the ability of SLS to improve the solubility of poorly soluble drugs by preparing amorphous solid dispersions [65,66].

In general, the influence of di-carboxylic acids and ibuprofen acid on the SOF’s properties could not be attributed to their plasticizing efficiency in SLS, as even non-plasticizing IbuNa and tartaric acid showed similar effects. Other factors were determinant for the properties of SOFs, such as the laser energy density, the compactness of the powder and the nature of the polymeric carrier. Amorphous copovidone already presents a low T_g_ compared to other polymers, which explains the low mechanical properties of the sintered SOFs [45]. Its low compactness is also involved in the reduction of the hardness [17]. Most importantly, its high solubility in water explains the rapid disintegration of the SOFs and the fast release of the drug [66].

### 3.5. Drug Degradation Evaluation

UHPLC analysis of the different SOFs printed with API revealed only one chromatographic peak corresponding to ibuprofen at the retention time (t_r_ = 1.50 min). In addition, the drug concentration in the different SOFs was analysed and it was found that the drug content was in agreement with the initial loading of the formulation (Table 5). This proves that no degradation has occurred during the sintering process, neither for the acid form nor for the sodium salt form. Therefore, no conclusion was possible regarding the presumed protective effect of SA as no degradation has been detected whether in absence or presence of the acid. As for IbuAc, it did not degrade despite its thermolability, suggesting a potential “autoprotective” effect due to its ability to decrease the optimal heating temperature. Further work will be needed using more thermolabile drugs without plasticizing properties in order to evidence the protective effect of succinic acid. 

### 3.6. Mechanisms of Plasticization in SLS

Although DSC and FTIR techniques have evidenced a decrease in the T_g_ and the presence of interactions between the components used in this study to sinter SOFs, they did not clearly discriminate between the different di-carboxylic acids as well as the two forms of ibuprofen. Nevertheless, hypotheses on the plasticization mechanisms can be formulated based on the chemical structure of the different excipients and drugs. 

Among the used di-carboxylic acids, succinic acid exhibited the highest plasticizing effect and allowed the heating temperature to be reduced by 15 °C. This shows that the carboxylic groups played a role in plasticization, presumably by establishing H-bonds with the C=O functions of copovidone [52,67,68]. The plasticizing efficiency of succinic acid increased with its percentage in the formulation. However, above 15% the optimal heating temperature remained the same. These results can be explained by either a potential phase separation occurring at high content of plasticizer which prevents the creation of interactions with the polymer [69]. Or another reason could be that above 15%, an excess of H-bonds is formed between the acid and the polymer which would reduce the mobility of the polymer chains instead of increasing it [44]. Furthermore, renewable materials already produced on an industrial scale such as succinic acid seem good candidates to replace current synthetic plasticizers [70]. 

When more rigidity was introduced into the structure of the di-carboxylic acid (fumaric and maleic acids), less effect was observed in lowering the optimal heating temperature compared to succinic acid (5 °C instead of 15 °C). It seems that introducing a double bond in the structure provides less flexibility for the plasticizers to interact with the copovidone [71]. Furthermore, switching from the conformation *trans* (fumaric acid) to the conformation *cis* (maleic acid) did not have an impact in processability. 

The effect of adding more hydroxyls into the structure while maintaining similar flexibility was studied by comparing succinic acid to malic acid and tartaric acid. In this case, the introduction of hydroxyl groups did not promote plasticization but instead reduced it, as previously seen with polyvinyl alcohol/starch films plasticized with polyols [46]. In this example, mannitol demonstrated inferior plasticizing properties than hexanetriol due to the presence of three additional hydroxyl groups in the structure, which increased its molecular weight and prevented its diffusion in the molecular matrix. Furthermore, the increased number of potential hydrogen bonds could also make the polymer structure more rigid and reduce the mobility of the chains [44]. This phenomenon is described as antiplasticization and is caused by a reduction of the free volume of the polymer by “filling the holes” with small molecules [72].

A noticeable effect on the processability was also observed when ibuprofen sodium salt was replaced by ibuprofen acid (a difference of 40 °C in OHT between KVA64 95%/IbuNa 5% and KVA64 95% / IbuAc 5%). A potential explanation for this difference in temperature is the formation of interactions in the forms prepared with copovidone and ibuprofen acid, as opposed to the salt form which do not exhibit interactions with the polymer [73]. Ibuprofen acid is known as a “non-traditional” plasticizer [39]. These non-traditional plasticizers are drugs that, in addition to improving the mechanical properties of films, could also provide a technical advantage by lowering the temperature during the hot-melt extrusion process [74]. As previously mentioned, IbuAc is poorly soluble in water and unstable at high temperature. However, during SLS its plasticizing effect may protect it from degradation and its potential amorphization during the sintering process can be an asset to enhance its solubility. As for IbuNa, replacing the hydrogen in the carboxylic function by sodium atoms blocks the plasticizing effect but enhances both solubility and thermal stability. Ibuprofen acid exhibited a higher plasticizing effect compared to succinic acid, despite its higher molecular weight and the presence of only one carboxylic group. This could be explained by the ability of IbuAc to establish aromatic bonds in addition to H-bonds with poly(vinylpyrrolidone) as previously reported by Bogdanova et al. [75]. This was observed in our previous work [17] with paracetamol, which also has an aromatic ring and acts as a plasticizer. 

## 4. Conclusions

This study represents a proof of concept that di-carboxylic acids can be used as potential plasticizers to decrease the optimal heating temperature for the selective laser sintering of pharmaceutical solid oral forms prepared with copovidone. Depending on the chemical structure of the acid, a different effect on processability was demonstrated. Succinic acid was identified as the most performant plasticizer, as it allows a higher decrease in the optimal heating temperature when incorporated. Furthermore, the model drug used in this work, ibuprofen, in its acid form acts as a non-traditional plasticizer by lowering the heating temperature considerably, opposed to the sodium salt ibuprofen which exhibited no influence on the process.

DSC analysis confirmed the plasticizing effect of the different excipients and drugs by evidencing a decrease in a glass transition temperature and FTIR analysis showed the presence of interactions between the polymer and the di-carboxylic acids or the drugs in the sintered solid oral forms. Both techniques displayed limits when discriminating between the different plasticizers. However, based on the chemical structure of the components, it was concluded that interactions such as hydrogen bonds promote plasticization, but an excess could inversely reduce the mobility of polymer chains. In the future, other techniques could be employed to provide more insight on the mechanisms of plasticization, such as FTIR at variable temperature and molecular dynamic simulations. 

Moreover, introduction of excipients and/or drugs in the formulation modified the properties (weight, hardness, disintegration time) of the solid oral forms, but this effect was more correlated to the decrease in the proportion of the polymer than the plasticization. Their beneficial effect on protecting the drug from degradation was not demonstrated since both forms of ibuprofen remained stable at the printing parameters. This suggests that these plasticizers aim more to facilitate the process than improving the properties of the printed solid oral forms. 

Overall, this work highlights the importance of understanding the relationship between the material properties and the process parameters, especially with innovative technologies such as selective laser sintering. Further work is encouraged on this research path with different polymers and more thermosensitive drugs, in order to explore more profoundly the advantages of using di-carboxylic acids in SLS. 

## Figures and Tables

**Table 1:** Composition of the different mixtures.

Mixtures	KVA64	IbuAc	IbuNa	SA	FA	MA	MLA	TA
**KVA64/IbuAc**	95%	5%						
**KVA64/IbuNa**	95%		5%					
**KVA64/SA**	95%			5%				
**KVA64/FA**	95%				5%			
**KVA64/MA**	95%					5%		
**KVA64/MLA**	95%						5%	
**KVA64/TA**	95%							5%
**KVA64/SA10**	90%			10%				
**KVA64/SA15**	85%			15%				
**KVA64/SA20**	80%			20%				
**KVA64/IbuNa/SA**	90%		5%	5%				
**KVA64/IbuNa/SA10**	85%		5%	10%				
**KVA64/IbuNa/SA15**	80%		5%	15%				
**KVA64/IbuNa/SA20**	75%		5%	20%				

KVA64: Kollidon VA64, IbuAc: ibuprofen acid, IbuNa: ibuprofen sodium salt, SA: succinic acid, FA: fumaric acid, MA: maleic acid, MLA: malic acid, TA: tartaric acid.

**Table 2:** Optimal heating temperatures for the different powders.

Powders	Optimal Heating Temperature (°C)
**KVA64**	110
**KVA64/IbuAc**	70
**KVA64/IbuNa**	110
**KVA64/SA**	95
**KVA64/FA**	105
**KVA64/MA**	105
**KVA64/MLA**	105
**KVA64/TA**	110
**KVA64/SA10**	85
**KVA64/SA15**	80
**KVA64/SA20**	80
**KVA64/IbuNa/SA**	95
**KVA64/IbuNa/SA10**	85
**KVA64/IbuNa/SA15**	80
**KVA64/IbuNa/SA20**	80

**Table 3:** Thermal properties of the different components and mixtures.

Powders	T_g_ (°C)	Tm (°C)	Td_2%_ (°C)
**KVA64**	99.30 ± 1.27	/	302.54
**IbuAc**	/	79.51	137.12
**IbuNa**	/	164.61	234.09
**SA**	/	190.42	166.86
**FA**	/	299.01	193.77
**MA**	/	146.65	142.15
**MLA**	/	106.67	165.79
**TA**	/	174.99	190.74
**KVA64/IbuAc**	80.69 ± 0.47	/	235.97
**KVA64/IbuNa**	93.15 ± 1.41	/	278.71
**KVA64/SA**	84.59 ± 0.86	/	237.92
**KVA64/FA**	84.25 ± 1.54	/	256.67
**KVA64/MA**	85.68 ± 0.24	/	207.91
**KVA64/MLA**	84.26 ± 1.35	/	239.32
**KVA64/TA**	89.55 ± 1.27	/	230.65
**KVA64/SA10**	74.40 ± 1.18	/	218.57
**KVA64/SA15**	66.84 ± 1.88	/	208.37
**KVA64/SA20**	57.51 ± 0.89	/	195.95
**KVA64/IbuNa/SA**	75.39 ± 1.29	/	237.82
**KVA64/IbuNa/SA10**	63.90 ± 0.34	/	217.75
**KVA64/IbuNa/SA15**	58.56 ± 1.77	/	197.90
**KVA64/IbuNa/SA20**	45.07 ± 1.05	/	186.50

T_g_: glass transition temperature, Tm: melting temperature, Td_2%_: temperature at 2% of degradation.

**Table 4:** Weight, hardness and disintegration time of the sintered SOFs.

Powders	Weight (mg)	Hardness (N)	Disintegration Time (s)
**KVA64**	153.0 ± 2.5	38.6 ± 4.5	73 ± 14
**KVA64/IbuAc**	132.2 ± 4.6	15.8 ± 3.8	24 ± 7
**KVA64/IbuNa**	170.8 ± 7.6	29.8 ± 2.5	58 ± 8
**KVA64/SA**	140.1 ± 2.1	25.6 ± 1.5	39 ± 4
**KVA64/FA**	149.1 ± 4.8	33.0 ± 1.7	43 ± 8
**KVA64/MA**	142.2 ± 1.8	24.2 ± 4.0	30 ± 8
**KVA64/MLA**	147.4 ± 1.1	31.9 ± 1.7	57 ± 9
**KVA64/TA**	166.0 ± 7.6	33.8 ± 4.2	43 ± 5
**KVA64/SA10**	138.4 ± 1.8	24.1 ± 2.3	25 ± 3
**KVA64/SA15**	139.2 ± 2.3	23.5 ± 3.0	21 ± 4
**KVA64/SA20**	138.9 ± 1.6	25.2 ± 2.4	23 ± 3
**KVA64/IbuNa/SA**	133.9 ± 1.7	16.6 ± 1.8	24 ± 3
**KVA64/IbuNa/SA10**	129.9 ± 1.4	16.5 ± 1.2	25 ± 3
**KVA64/IbuNa/SA15**	131.3 ± 2.5	18.8 ± 1.8	34 ± 5
**KVA64/IbuNa/SA20**	128.9 ± 1.7	18.0 ± 1.0	30 ± 6

**Table 5:** Drug content of the different SOFs printed with drugs.

Formulation	Drug Content (%)
**KVA64/IbuAc**	102.2 ± 2.0
**KVA64/IbuNa**	96.5 ± 2.7
**KVA64/IbuNa/SA**	103.0 ± 4.1
**KVA64/IbuNa/SA10**	102.8 ± 3.6
**KVA64/IbuNa/SA15**	104.1 ± 4.5
**KVA64/IbuNa/SA20**	103.0 ± 2.2

## Data Availability

Data is contained within the article or supplementary material.

## Supplementary Materials

The following are available online at https://www.mdpi.com/article/10.3390/polym13193282/s1, Figure S1: DSC thermograms of KVA64 during the 1st and 2nd heatings. Figure S2: DSC thermogram of IbuNa. Figure S3. Images of the mixtures KVA64 95%/SA 5% and KVA64 95%/TA 5%: (a) SEM images of the powders (prior to sintering) (Magnification × 100), (b) SEM images of the SOFs vertical sections (Magnification × 35), images of the SOFs. Table S1: Bulk density and true density of the different printed powders.
