# Supplementary material for: Investigating the Potential Plasticizing Effect of Di-Carboxylic Acids for the Manufacturing of Solid Oral Forms with Copovidone and Ibuprofen by Selective Laser Sintering
Source: Polymers (Basel). 2021 Sep 26;13(19):3282. doi: 10.3390/polym13193282 (PMC8513101; doi:10.3390/polym13193282)
Supplement: Supplementary file 1 [file polymers-13-03282-s001.zip › polymers-1381547-supplementary.pdf]

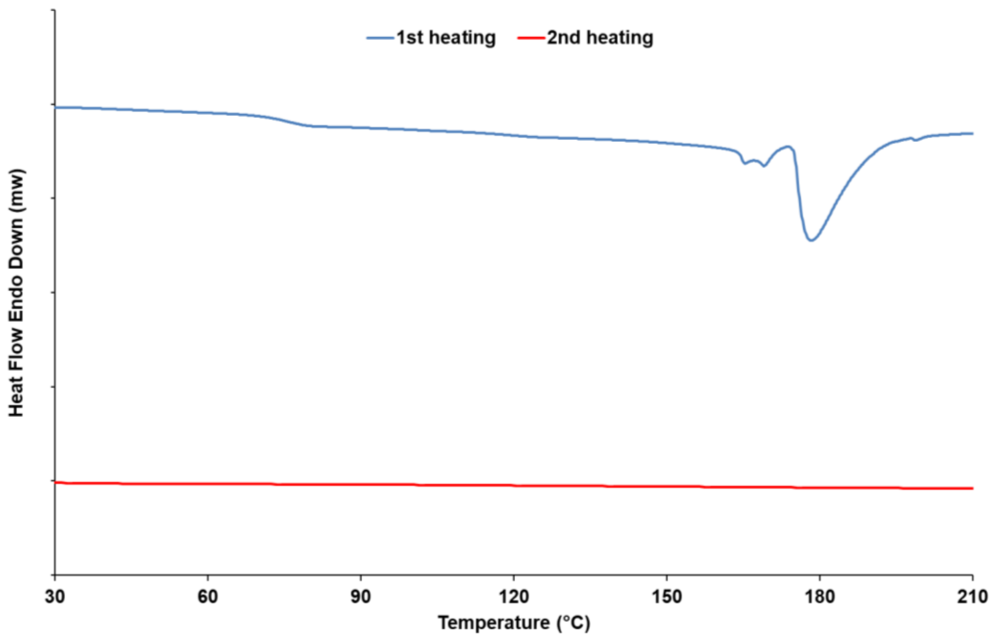

Figure S1. DSC thermograms of KVA64 during the 1st and 2nd heatings.

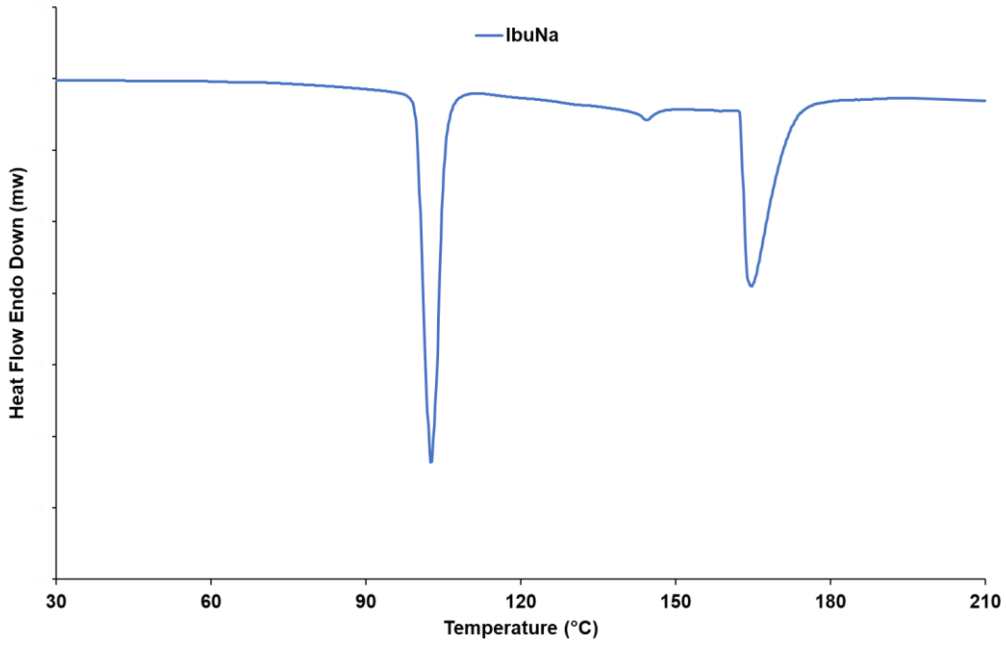

Figure S2. DSC thermogram of IbuNa.

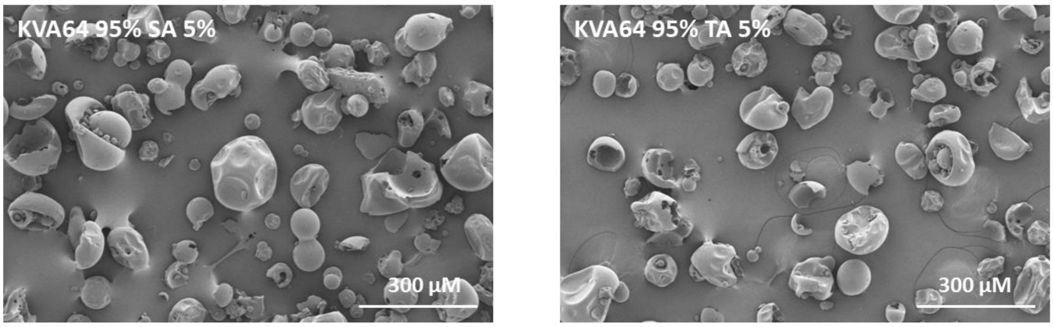

(a)

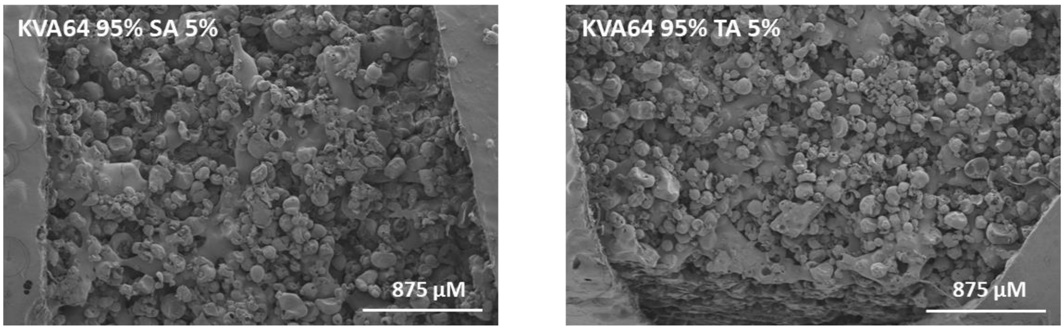

(b)

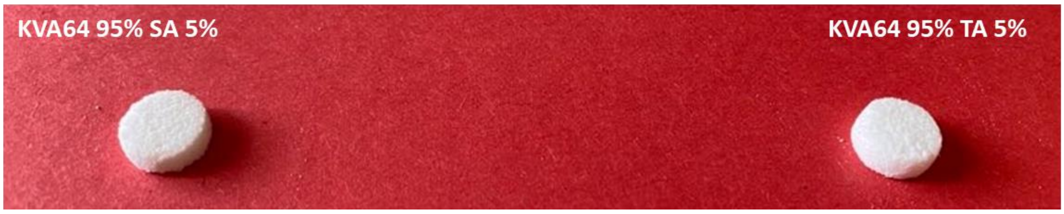

(c)

Figure S3. Images of the mixtures KVA64 95% / SA 5% and KVA64 95% / TA 5%: (a) SEM images of the powders (prior to sintering) (Magnification x 100), (b) SEM images of the SOFs vertical sections (Magnification x 35), images of the SOFs.

Table S1. Bulk density and true density of the different printed powders.

Powders	Bulk density (g/cm^3)	True density (g/cm^3)
**KVA64**	0.38 ± 0.00	1.23 ± 0.01
**KVA64/IbuAc**	0.32 ± 0.00	1.18 ± 0.02
**KVA64/IbuNa**	0.37 ± 0.01	1.19 ± 0.00
**KVA64/SA**	0.38 ± 0.00	1.21 ± 0.00
**KVA64/FA**	0.37 ± 0.01	1.21 ± 0.00
**KVA64/MA**	0.40 ± 0.00	1.20 ± 0.01
**KVA64/MLA**	0.38 ± 0.01	1.20 ± 0.00
**KVA64/TA**	0.40 ± 0.01	1.20 ± 0.00

**KVA64/SA10**	0.38 ± 0.00	1.21 ± 0.01
**KVA64/SA15**	0.38 ± 0.00	1.22 ± 0.02
**KVA64/SA20**	0.39 ± 0.00	1.25 ± 0.00
**KVA64/IbuNa/SA**	0.37 ± 0.00	1.20 ± 0.00
**KVA64/IbuNa/SA10**	0.37 ± 0.00	1.20 ± 0.02
**KVA64/IbuNa/SA15**	0.38 ± 0.00	1.23 ± 0.00
**KVA64/IbuNa/SA20**	0.39 ± 0.01	1.25 ± 0.00
